# Myeloid nuclear differentiation antigen, neutrophil apoptosis and sepsis

**DOI:** 10.3389/fimmu.2012.00397

**Published:** 2012-12-27

**Authors:** Eric Milot, Nasser Fotouhi-Ardakani, János G. Filep

**Affiliations:** ^1^Department of Medicine, Maisonneuve-Rosemont Hospital Research Center, University of MontréalMontréal, QC, Canada; ^2^Department of Pathology and Cell Biology, Maisonneuve-Rosemont Hospital Research Center, University of MontréalMontréal, QC, Canada

**Keywords:** MNDA, sepsis, neutrophils, MCL-1, mitochondria, internal apoptosis pathway, inflammation

## Abstract

Sepsis and septic shock are characterized by prolonged inflammation and delayed resolution, which are associated with suppression of neutrophil apoptosis. The role of the intrinsic apoptotic pathway and intracellular factors in regulation of neutrophil apoptosis remain incompletely understood. We previously reported that the nuclear factor MNDA (myeloid nuclear differentiation antigen) is fundamental to execution of the constitutive neutrophil death program. During neutrophil apoptosis MNDA is cleaved by caspases and relocated to the cytoplasm. However, when challenged with known mediators of sepsis, human neutrophils of healthy donors or neutrophils from patients with sepsis exhibited impaired MNDA relocation/cleavage parallel with myeloid cell leukemia-1 (MCL-1) accumulation and suppression of apoptosis. MNDA knockdown in a model cell line indicated that upon induction of apoptosis, MNDA promotes proteasomal degradation of MCL-1, thereby aggravating mitochondrial dysfunction. Thus, MNDA is central to a novel nucleus-mitochondrion circuit that promotes progression of apoptosis. Disruption of this circuit contributes to neutrophil longevity, thereby identifying MNDA as a potential therapeutic target in sepsis and other inflammatory pathologies.

## INTRODUCTION

Different types of hematopoietic cells participate in the inflammatory response to microbial infection. Among them, circulating neutrophils are rapidly recruited into infected or injured tissues. They are the first line of defense against pathogens and are key regulators of the initial response to microbial infection. Effective removal of neutrophils from inflamed tissues is critical for timely resolution of inflammation. However, because of the disruption of neutrophil programmed cell death in inflammatory-related conditions, including sepsis, neutrophils persist in tissues and blood and portend poor prognosis. Here, we will discuss the recent discovery of a novel nuclear to mitochondrion circuit that is involved in the control of neutrophil apoptosis and disrupted during sepsis.

## SEPSIS

Sepsis and septic shock (hereafter commonly referred as sepsis) are portent major medical challenges that result from a harmful host response to infection. Sepsis has a high prevalence and morbidity. At the beginning of this century, Angus et al. (2001) reported that, at the time, 9.3% of all cases of death in the USA was caused by sepsis. The incidence of sepsis was then evaluated as 3 cases per 1000 people, and 2.26 cases per 100 hospital-discharged patients. The mortality was estimated at 26.6% of all sepsis cases but, this percentage was significantly higher with elderly patients. The prevalence of this disease is increasing year after year despite advances in critical care. It is now considered to be the 10th leading cause of mortality in the United States (Melamed and Sorvillo, 2009).

Sepsis results from an inappropriate host response to infection. The initial stage of sepsis is usually considered to result from an exaggerated or dysregulated inflammatory response to infection (Pene et al., 2012). As sepsis persists, a shift toward immunosuppression is observed (Hotchkiss and Nicholson, 2006), concomitant with occurrence of organ failure and secondary infection. The severity of sepsis is frequently evaluated by various scoring systems, including the APACHE II (Acute Physiology and Chronic Health Evaluation II; Knaus et al., 1985) or SAPS II (Simplified Acute Physiology Score II; Le Gall et al., 1993) within the first 24 h of hospitalization. The score is based on measurements of vital parameters such as blood pressure, heart rate, respiratory rate, temperature, neutrophil count, etc. Intriguingly, both high and low blood neutrophil counts (neutrophilia and neutropenia, respectively) portend poor prognosis. Despite extensive efforts, specific molecular markers for identifying patients with high risk for sepsis or its more severe form, septic shock, have not been identified. Molecular markers with limited accuracy and specificity have been proposed for defining the stages of the disease. For instance, the prototypic acute-phase reactant C-reactive protein can be used as a marker of systemic inflammation during sepsis, whereas high-levels of procalcitonin are detectable at early stage of bacterial infection (Aalto et al., 2004).

## NEUTROPHILS AND SEPSIS

Neutrophils are the first line of defense against pathogens. They generate different proteolytic enzymes as well as reactive oxygen species (ROS) and reactive nitrogen species (RNS) to destroy invading microorganisms following phagocytosis, or extracellulary by neutrophil extracellular trap (NET) formation (Papayannopoulos et al., 2010; Metzler et al., 2011).

Neutrophils have the shortest life span among leukocytes and undergo constitutive programmed cell death (apoptosis). This process is essential for regulation of neutrophil homeostasis. Constitutive apoptosis renders neutrophils unresponsive to extracellular stimuli and allows their recognition and removal by macrophages (Savill et al., 2002; Gilroy et al., 2004). This process is critical for termination of the inflammatory response and tissue repair. Following discharging their function, at the inflammatory locus, extravasated neutrophils are though to predominantly undergo apoptosis. However, signals from the inflammatory milieu can either accelerate or suppress the cell death program, thereby affecting the fate of neutrophils (Gilroy et al., 2004). Suppressed neutrophil apoptosis is often detected in patients with inflammatory pathologies, including sepsis and septic shock and portends poor prognosis (Keel et al., 1997; Matute-Bello et al., 1997; Taneja et al., 2004). Exposure of neutrophils to inflammatory mediators such as GM-CSF, IL-8 or to bacterial constituents results in delayed apoptosis (El Kebir and Filep, 2010; Geering and Simon, 2011). Preserving neutrophil activities at the sites of infection may be required for complete elimination of invading pathogens, but could also aggravate injury to the host, resulting in persistent tissue damage. Therefore, the regulation of neutrophil apoptosis is critical to control the balance between their antimicrobial effectiveness and potential deleterious effect on tissues.

Signaling pathways promoting survival of neutrophils during sepsis are converging to control expression and degradation of key factors influencing the programmed cell death. In mature neutrophils, the anti-apoptotic protein myeloid cell leukemia-1 (MCL-1) and the pro-apoptotic protein Bcl2-associated X (BAX) are critical for the regulation of mitochondrial transmembrane potential (ΔΨ_m_), and hence, activation of effector caspases (El Kebir and Filep, 2010; Geering and Simon, 2011; Milot and Filep, 2011). Since the control of mitochondrial transmembrane potential is central to the intrinsic apoptotic pathway, these discoveries placed forth the intrinsic apoptotic pathway in regulation of neutrophil apoptosis.

## INTRINSIC APOPTOSIS PATHWAY AND MCL-1 IN NEUTROPHILS

MCL-1 is an anti-apoptotic factor of the Bcl-2 family. MCL-1 accumulation protects against formation of the BAK-BAX heterodimer on the external mitochondrial membrane and subsequent release of cytochrome *c* along with other molecules influencing apoptosis like SMAC/Diablo, endonuclease G, and AIF (apoptosis-inducing factor), from the mitochondrial inner membrane. Hence, MCL-1 protects ΔΨ_m_ and thus regulates the internal apoptotic pathway.

Unlike other members of the Bcl-2 family, MCL-1 protein has a short half-life and its levels of expression change substantially as neutrophils age and upon exposure of neutrophils to inflammatory mediators (Moulding et al., 2001; Craig, 2002). Indeed, MCL-1 protein expression inversely correlates with the degree of neutrophil apoptosis in both experimental models and clinical settings. Rapid loss in MCL-1 corresponds to development of apoptosis and MCL-1 knockdown results in dramatic decreases in the neutrophil lifespan (Moulding et al., 1998; Dzhagalov et al., 2007). Modification in *Mcl-1* transcription accounts for most variation of MCL-1 expression observed upon stress conditions (Dong et al., 2011). At the transcription level, *Mcl-1* is regulated by different transcription factors including MYC, NF-κB (RelA/p65), STAT5, and HIF-1α (Akgul et al., 2000; Negrotto et al., 2006; Beverly and Varmus, 2009; Thomas et al., 2010). RNA processing and protein accumulation/turnover are also important for regulation of MCL-1 expression (Bae et al., 2000). The turnover of MCL-1 results primarily from the proteasome activity (Zhong et al., 2005). MULE/Arf-BP1, an E3 ubiquitin ligase, ubiquitinates MCL-1 and subsequently enhances its proteasomal degradation (Zhong et al., 2005). This activity can be counterbalanced by the activity of the deubiquitinase USP9X which was demonstrated to deubiquitinate and thereby, to stabilize MCL-1 (Schwickart et al., 2010). However, surprisingly little is known about regulation of MCL-1. We have identified myeloid nuclear differentiation antigen (MNDA) as a regulator of the proteasomal degradation of MCL-1 (Fotouhi-Ardakani et al., 2010 and see below).

## ROLE OF MITOCHONDRIA IN NEUTROPHIL APOPTOSIS

In neutrophils, mitochondria have an atypical function and their role seems to be restricted to apoptosis (van Raam and Kuijpers, 2009). This view has been nourished by the observation that neutrophils rely on glycolysis for energy formation and even for a long time mitochondria could not be detected in these cells. The electron transport chain is inefficient to transport electrons from complexes III to IV in neutrophils (van Raam et al., 2008). However, it is not to say that it exerts no activity in neutrophils since, inhibitors of the mitochondrial respiratory chain complex I can modulate the severity of lung injury evoked by LPS (Zmijewski et al., 2009). Enhanced production of H_2_O_2_ by neutrophils results in inhibition of IκB-α degradation hence preventing the activation of NF-κB, a key regulator of inflammatory gene expression in neutrophils (Zmijewski et al., 2008). Thus, the mitochondrial respiratory chain appears to be only partially active in neutrophils.

## MNDA: A KEY COMPONENT OF A NOVEL NUCLEUS TO MITOCHONDRION CIRCUIT

Different factors exerting their activity in the nucleus have been reported to participate in and influence the internal apoptosis pathway. While some nuclear proteins including E2F1, STAT3, HIF-1α, and NF-κB are well known to regulate expression of genes encoding pro- or anti-apoptotic factors, other nuclear proteins like MNDA, p53, p21/WAF1, proliferating cell nuclear antigen (PCNA), nur77, SHP, and possibly p73, have been reported or proposed to act as nuclear signals (transducers) to influence the intrinsic apoptotic pathway upon relocation or specific cytoplasmic accumulation (Chipuk et al., 2003; Dumont et al., 2003; Mihara et al., 2003; Wang, 2005; Fotouhi-Ardakani et al., 2010; Witko-Sarsat et al., 2010; Milot and Filep, 2011). Some of these factors have been reported to directly affect pro- or anti-apoptotic factors and hence, apoptosis. MNDA is one of them.

Myeloid nuclear differentiation antigen is a human hematopoietic specific factor of the HIN-200 family. This family of factors is composed of the functionally related proteins IFI16, AIM2, IFIX, and MNDA (Choubey and Panchanathan, 2008). MNDA localizes predominantly to the nucleus and is expressed mainly in myeloid cells. It has been suggested that MNDA may function as a master regulator of monocytic and granulocytic lineages (Novershtern et al., 2011). Recently, MNDA has been proposed to be a transcription factor (Suzuki et al., 2012). Like other members of the HIN-200 family, MNDA contains a pyrin/PAAD/DAPIN domain that mediates binding between proteins involved in apoptotic and inflammatory signaling pathways (Fairbrother et al., 2001). It also contains a HIN-200 domain, which is thought to promote protein–protein (Dawson and Trapani, 1996; Choubey and Panchanathan, 2008) and protein–DNA interactions (Jin et al., 2012). *MNDA* gene regulation is influenced by interferons (Choubey and Panchanathan, 2008). MNDA was initially proposed to regulate myeloid cell differentiation as well as development of sporadic myelodysplastic syndrome (Briggs et al., 2006).

The potential implication of MNDA in regulation of apoptosis in myeloid cells and in inflammation has been directly assessed in neutrophil granulocytes (Fotouhi-Ardakani et al., 2010). In bone marrow-derived and mature neutrophils, MNDA is predominantly located in the nucleus. In neutrophils undergoing apoptosis, MNDA is cleaved by caspases, presumably caspase-3, and relocated to the cytoplasm. However, the cleavage of MNDA is likely not required for its cytoplasmic accumulation since the full-length MNDA could also be detected in the cytoplasm. Culture of human neutrophils with inflammatory mediators, like bacterial constituents and platelet-activating factor, promotes their survival and indicates a clear correlation between the degree of neutrophil apoptosis and MNDA cleavage as well as cytoplasmic accumulation. These findings suggest that MNDA could participate in regulation of apoptosis in neutrophils.

A causal relationship between MNDA and apoptosis has been established in a model cell line, the promyelocytic leukemia cell line HL-60, which expresses endogenous MNDA (Duhl et al., 1989; Savli et al., 2002). We created two MNDA-deficient HL-60 cell lines by the stable genomic integration of vectors encoding specific small hairpin RNA (shRNA). In these engineered model cell lines, knockdown of MNDA partially protected HL-60 cells against genotoxic stress-induced apoptosis, markedly attenuated activation of caspase-3, but not caspase-8, and prevented mitochondrial dysfunction (Fotouhi-Ardakani et al., 2010). These observations identify MNDA as a modulator of the intrinsic (mitochondrial) pathway of apoptosis.

**FIGURE 1:**
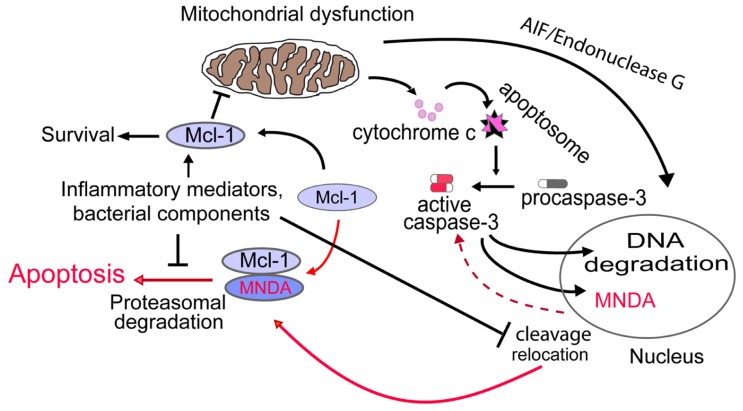
**Proposed model for MNDA regulation of neutrophil apoptosis**. Cytoplasmic relocation and cleavage of MNDA results in aggravation of mitochondrial dysfunction through promotion of proteasomal degradation of MCL-1. Activation of this novel nucleus-mitochondrion circuit would then accelerate execution of the apoptotic death program. Conversely, prevention of MNDA relocation and cleavage would prolong neutrophil survival by retarding apoptosis. The mechanisms by which bacterial components and/or inflammatory modulators could negatively influence MNDA relocation and cleavage and hence, interfere with this nuclear-mitochondrial circuit remains to be defined. Broken line indicates yet undefined mechanism.

The importance of the anti-apoptotic factor MCL-1 in control of ΔΨ_m_ and neutrophil apoptosis (Moulding et al., 1998; Dzhagalov et al., 2007) led us to interrogate whether MNDA could influence the internal pathway of apoptosis via MCL-1. Interestingly, we found that: (i) MNDA co-immunoprecipitates with MCL-1; and (ii) after induction of apoptosis, MCL-1 accumulation was greatly enhanced in MNDA-deficient HL-60 cells compared to MNDA proficient HL-60 cells (Fotouhi-Ardakani et al., 2010). Similar results were obtained in the presence of the protein synthesis inhibitor cycloheximide, suggesting that MNDA influences the turnover of MCL-1 protein. Since MCL-1 turnover is mainly regulated by proteasomal degradation (Zhong et al., 2005), we blocked the proteasome activity with MG132 and found that under such condition, MNDA failed to affect MCL-1 accumulation. These findings confirm that the rapid fall in MCL-1 expression is due to proteasomal degradation and indicate that, when present, MNDA promotes proteasomal degradation of MCL-1. By contrast, MNDA knockdown slowed down MCL-1 turnover and rendered HL-60 cells resistant to genotoxic stress-induced apoptosis, indicating that MNDA regulation of MCL-1 degradation is required for the execution of the constitutive cell death program. Collectively these findings indicate that cytoplasmic accumulation of MNDA is not merely a consequence, but rather an important mechanism promoting apoptosis in HL-60 cells and likely, in mature human neutrophils (**Figure 1**).

It is not known whether co-immunoprecipitation of MNDA and MCL-1 resulted from direct protein–protein interaction or which region(s) of MNDA is(are) required for this association. However, the MNDA PAAD/DAPIN/Pyrin domain, which is common to different proteins involved in apoptosis and inflammation, and/or the HIN-200 domain that mediates protein–protein interactions (Asefa et al., 2004) could be critical for the MNDA interaction with MCL-1. Indeed, the PAAD/DAPIN/Pyrin domain was shown to promote self-association of MNDA (Xie et al., 1997), and might also mediate association with other proteins. For instance, IFI16, which contains a PAAD/DAPIN/Pyrin domain, interacts with p53, thereby modulating senescence and apoptosis (Song et al., 2008). In mice, members of the HIN-200 family were shown to promote inflammation through interacting with NF-κB (Min et al., 1996). These results demonstrate that a member of the HIN-200 family or a protein with the PAAD/DAPIN/Pyrin domain co-immunoprecipitates with an anti-apoptotic protein of the Bcl-2 family to regulate apoptosis. It remains to be investigated whether this mechanism is common to all MNDA expressing cells including hematopoietic progenitors (Briggs et al., 2006).

## ROLE FOR MNDA DURING SEPSIS

It is well established that neutrophils isolated from the peripheral blood of healthy volunteers undergo apoptosis when cultured for 24–48 h *in vitro*. By contrast, under the same conditions of culture, neutrophils of patients with sepsis exhibit markedly prolonged survival due to suppressed apoptosis (Keel et al., 1997; Matute-Bello et al., 1997; Fotouhi-Ardakani et al., 2010; Paunel-Gorgulu et al., 2012). The enhanced neutrophil longevity is associated with preserved ΔΨ_m_ and inversely correlates with cytoplasmic accumulation of MNDA (Fotouhi-Ardakani et al., 2010). As predicted from the comprehensive study on MNDA in model cell lines (see above), during neutrophil apoptosis MNDA is relocated from the nucleus to the cytoplasm whereby it directly interacts with MCL-1 and promotes its proteasomal degradation (Figure 1). Although the signaling pathways involved in these events have not been elucidated, MNDA remains sequestered in the nucleus of neutrophils of patients in sepsis (Fotouhi-Ardakani et al., 2010). Consistently, culture of neutrophils from healthy volunteers with LPS, bacterial DNA, or platelet-activating factor partially replicated the abnormalities seen in the clinical samples, including the sequestration of MNDA to the nucleus (Fotouhi-Ardakani et al., 2010). Most interestingly, similar results were obtained when neutrophils of healthy donors were cultured in presence of serum from sepsis patient (Fotouhi-Ardakani et al., 2010). These findings suggest that neutrophils integrate yet unidentified cues from the inflammatory milieu, which would prevent the cytoplasmic relocation and/or accumulation of MNDA, events that favor neutrophil apoptosis. Although our pilot clinical study was not powered to assess outcome, we noted that patients who had died exhibited markedly suppressed neutrophil apoptosis with minimal or complete absence of MNDA translocation and/or cleavage in neutrophils. Suppressed apoptosis in circulating neutrophils may contribute to neutrophilia, which predicts a poor prognosis, whereas delayed apoptosis in emigrated or trapped neutrophils contributes to aggravation of tissue injury, in particular damage to the airways (Matute-Bello et al., 1997; Hotchkiss and Nicholson, 2006). Apoptotic neutrophils sequester cytokines during endotoxin shock in mice (Ren et al., 2008) and thus may contribute to resolution of sepsis. Conversely, failure of neutrophils to undergo timely apoptosis would likely impair this pro-resolution effect. Clearly, additional studies are required to assess the precise role of MNDA in facilitating resolution of inflammation.

In conclusion, cytoplasmic accumulation of MNDA plays an important role in the progression of apoptosis. This represents a novel mechanism whereby MNDA, which predominantly localizes to the nucleus, regulate MCL-1 degradation and consequently mitochondrial function following its accumulation in the cytoplasm. The investigation of MNDA in neutrophils demonstrates that prevention of cytoplasmic MNDA accumulation likely contributes to suppressed apoptosis of neutrophils in patients with sepsis. Therefore, targeting MNDA may have a therapeutic potential for the treatment of sepsis and other inflammatory disorders.

## Conflict of Interest Statement

The authors declare that the research was conducted in the absence of any commercial or financial relationships that could be construed as a potential conflict of interest.
